# Karyomorphometric Differentiation and Karyotype Asymmetry in Five Rare *Zingiber* Species from Thailand

**DOI:** 10.3390/plants15182740

**Published:** 2026-09-08

**Authors:** Piyaporn Saensouk, Surapon Saensouk, Rattanavalee Senavongse, Thawatphong Boonma, Kamonwan Koompoot, Pramote Triboun

**Affiliations:** 1 Department of Biology, Faculty of Science, Mahasarakham University, Maha Sarakham 44150, Thailand; pcornukaempferia@yahoo.com; 2 Diversity of Family Zingiberaceae and Vascular Plant for Its Applications Research Unit, Mahasarakham University, Maha Sarakham 44150, Thailand; boonma.thawat@gmail.com (T.B.); kamonwan.k@kkumail.com (K.K.); 3 Walai Rukhavej Botanical Research Institute, Mahasarakham University, Maha Sarakham 44150, Thailand; 4 School of Crop Production Technology, Institute of Agricultural Technology, Suranaree University of Technology, Nakhon Ratchasima 30000, Thailand; araceaefamily@gmail.com; 5 National Biobank of Thailand, National Science and Technology Development Agency, 111, Thailand Science Park, Khlong 1, Khlong Luang, Pathum Thani 12120, Thailand; tribountree@gmail.com

**Keywords:** karyomorphometry, karyotype asymmetry, centromeric index, chromosome number, *Zingiber*, Zingiberaceae

## Abstract

Cytogenetic information remains limited for many rare and geographically restricted species of *Zingiber* in Thailand. This study investigated chromosome numbers, karyotype characteristics, centromeric-index and relative chromosome-length variation, and karyotype asymmetry in *Z. brachystachys*, *Z. gramineum*, *Z. mekongense*, *Z. pellitum*, and *Z. pyroglossum*. Somatic chromosomes were examined from root-tip meristems using the aceto-orcein squash technique, followed by quantitative karyotype analysis, principal component analysis (PCA), UPGMA clustering, and Stebbins asymmetry classification. All five examined taxa had 2*n* = 22 and NF = 44 but differed in karyotype formula: 8sm + 3st, 3m + 7sm + 1st, 5sm + 6st, 1m + 5sm + 5st, and 7sm + 3st + 1t, respectively. Multivariate analyses incorporating both centromeric index (CI) and relative chromosome length (RL) showed clear karyomorphometric differentiation among the five taxa. UPGMA analysis grouped *Z. pellitum* and *Z. brachystachys* most closely, whereas *Z. gramineum* joined the remaining taxa at the greatest clustering distance. The first two PCA components explained 74.591% of the total variation. Stebbins categories ranged from 3A to 4B. These results provide new comparative cytogenetic data for five rare Thai *Zingiber* species and demonstrate interspecific variation in chromosome morphology despite uniformity in chromosome number.

## 1. Introduction

The ginger family, Zingiberaceae, is one of the most diverse families of tropical monocotyledons, comprising 59 accepted genera and approximately 2000 species worldwide [1]. Members of the family are predominantly perennial, rhizomatous herbs distributed throughout tropical and subtropical regions, with the greatest diversity occurring in South and Southeast Asia [2,3,4,5,6]. They occur in a wide range of habitats, including evergreen and seasonally dry forests, limestone areas, forest margins, and human-managed landscapes [6,7]. Many species are used as foods, spices, medicines, and ornamentals and are important components of local traditions and livelihoods [8,9].

Thailand is an important centre of Zingiberaceae diversity in mainland Southeast Asia. Floristic investigations in the Southwestern, Central, and Southeastern regions of the country have documented a rich ginger flora in both natural and human-managed habitats [6,9,10]. A recent province-level study has recorded high species richness, numerous endemic or geographically restricted taxa, and many previously undocumented provincial records [9]. These inventories also provide well-documented plant material for further taxonomic, ecological, conservation, and cytological studies.

The genus *Zingiber* Mill. belongs to tribe Zingibereae of subfamily Zingiberoideae and is distributed mainly in tropical and subtropical Asia [1,2,3,4]. The current treatment in the Flora of Thailand recognizes 58 species in the country [6], making *Zingiber* one of the most species-rich genera of Thai Zingiberaceae. Although the taxonomy and molecular relationships of *Zingiber* have received increasing attention [6,7,11,12,13], cytogenetic information remains limited for many wild species, particularly rare and geographically restricted taxa.

Cytogenetic characters, including somatic chromosome number, basic chromosome number, ploidy level, chromosome size, and karyotype structure, provide useful information for taxonomic and comparative studies in Zingiberaceae [14,15,16,17,18,19]. Previous cytological studies have generally regarded *x* = 11 as the basic chromosome number in *Zingiber* [14,15,16,17,18,19]. A somatic chromosome number of 2*n* = 22 has frequently been reported in the genus, including in *Z. officinale* Roscoe, *Z. zerumbet* (L.) Roscoe ex Sm., and several species from Thailand [20,21,22]. Nevertheless, chromosome-number variation also occurs within the genus. In *Z. mioga* (Thunb.) Roscoe, for example, chromosome numbers of 2*n* = 22, 44, and 55 have been reported from different geographical populations [23], corresponding to different ploidy levels based on *x* = 11. These records indicate that variation in chromosome complement may occur both among and within species of *Zingiber*.

Chromosome number alone, however, provides only part of the cytological information available for comparison among taxa. Species with the same somatic chromosome number may differ in chromosome size, relative chromosome length, arm ratio, centromere position, and overall karyotype composition. Quantitative karyotype analysis can therefore reveal differences that are not apparent from chromosome counts alone. In evaluating karyotype asymmetry, variation in chromosome length among chromosomes and variation in centromere position represent different components of karyotype structure and should be considered separately [24]. Multivariate analyses based on quantitative chromosome characters can also be used to summarize patterns of similarity and differentiation among taxa. Such patterns represent phenetic relationships based on the chromosome characters examined and should not be interpreted directly as phylogenetic relationships.

Despite the high diversity of *Zingiber* in Thailand, detailed cytogenetic information remains limited for many rare and geographically restricted species. Five species, *Z. brachystachys* Triboun & K.Larsen, *Z. gramineum* Noronha ex Blume, *Z. mekongense* Gagnep., *Z. pellitum* Gagnep., and *Z. pyroglossum* Triboun & K.Larsen, were therefore selected for cytogenetic investigation. Among these, *Z. brachystachys* is endemic to Thailand [1,6]. Published cytogenetic information on these taxa is limited, and comparative quantitative data on their karyotypes remain insufficient.

The present study aimed to determine the somatic chromosome numbers of these five *Zingiber* species, characterize their karyotypes using quantitative chromosome measurements, compare interspecific variation in chromosome size and centromere position, and assess patterns of cytogenetic similarity using multivariate ordination and hierarchical clustering. The results were also compared with previously published chromosome records for *Zingiber* and other members of tribe Zingibereae to provide additional cytogenetic information for the genus in Thailand.

## 2. Results

### 2.1. Chromosome Complement and Karyomorphometric Variation

**Figure 1:**
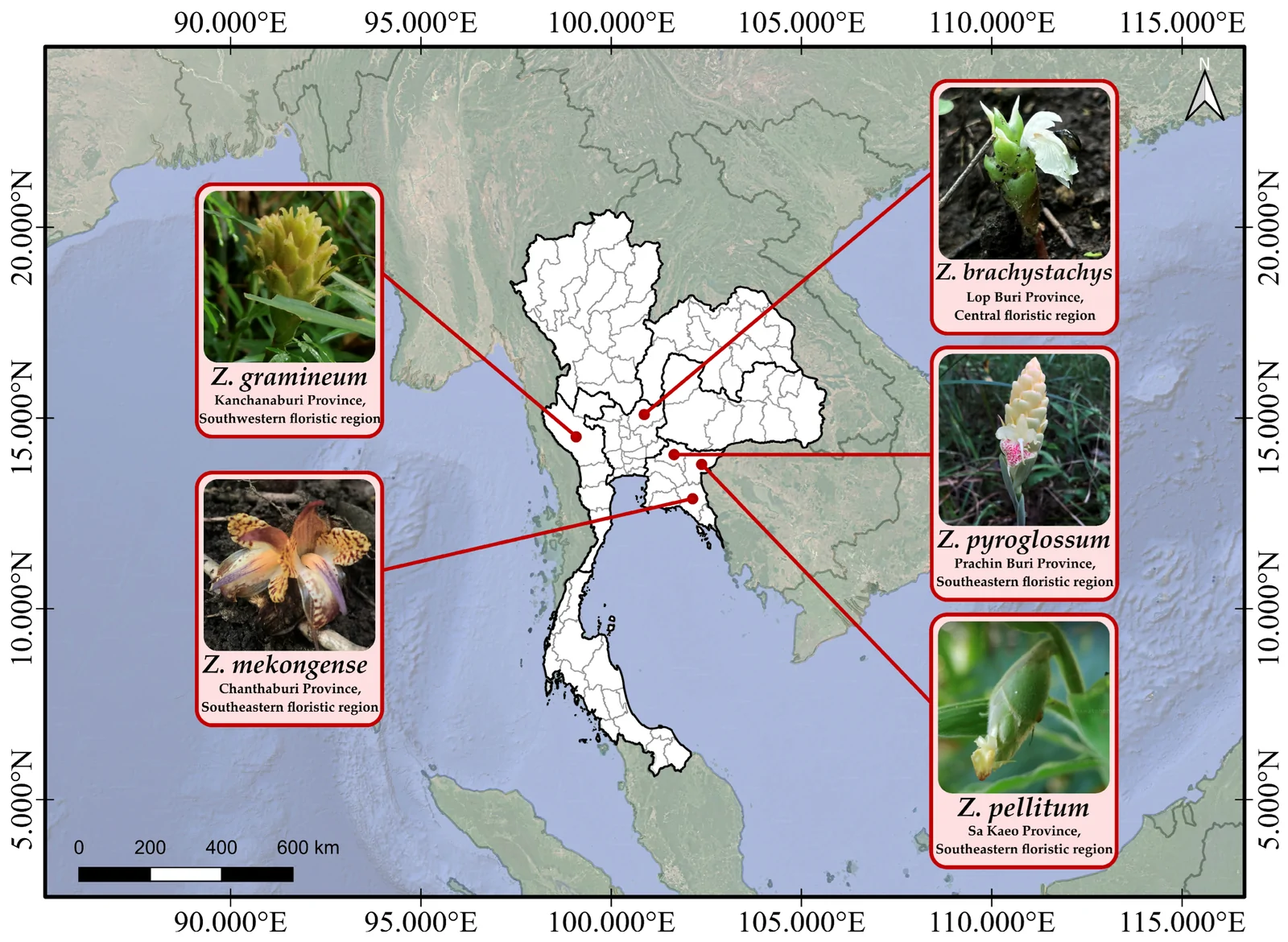
Collection localities and representative photographs of the five *Zingiber* species examined in this study in Thailand. Base map provided by K.K.; photographs of the five taxa were taken by T.B.

The five *Zingiber* species examined in this study, *Z. brachystachys*, *Z. gramineum*, *Z. mekongense*, *Z. pellitum*, and *Z. pyroglossum*, were collected from different provinces in the Central, Southwestern, and Southeastern floristic regions of Thailand (Figure 1). *Zingiber brachystachys* is endemic to Thailand, whereas the other four species are native to Thailand. Among the five species, *Z. gramineum*, *Z. pellitum*, and *Z. pyroglossum* are currently assessed as Least Concern [25,26,27], whereas *Z. brachystachys* and *Z. mekongense* have not been evaluated.

**Figure 2:**
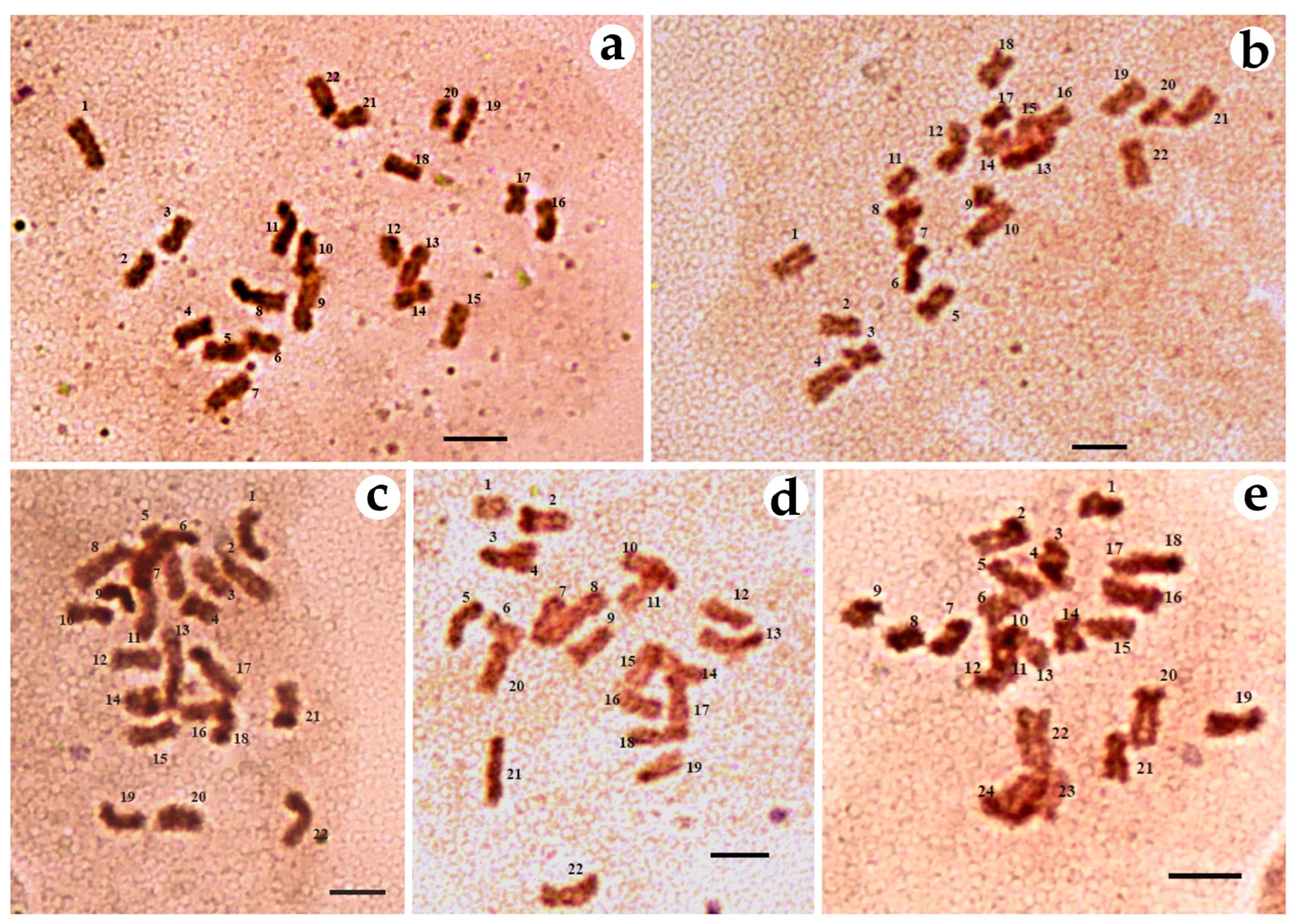
Photomicrographs of somatic metaphase chromosomes 2*n* = 22: (**a**) *Z. brachystachys*, (**b**) *Z. gramineum*, (**c**) *Z. mekongense*, (**d**) *Z. pellitum* and (**e**) *Z. pyroglossum* (Scale bars = 10 µm).

**Figure 3:**
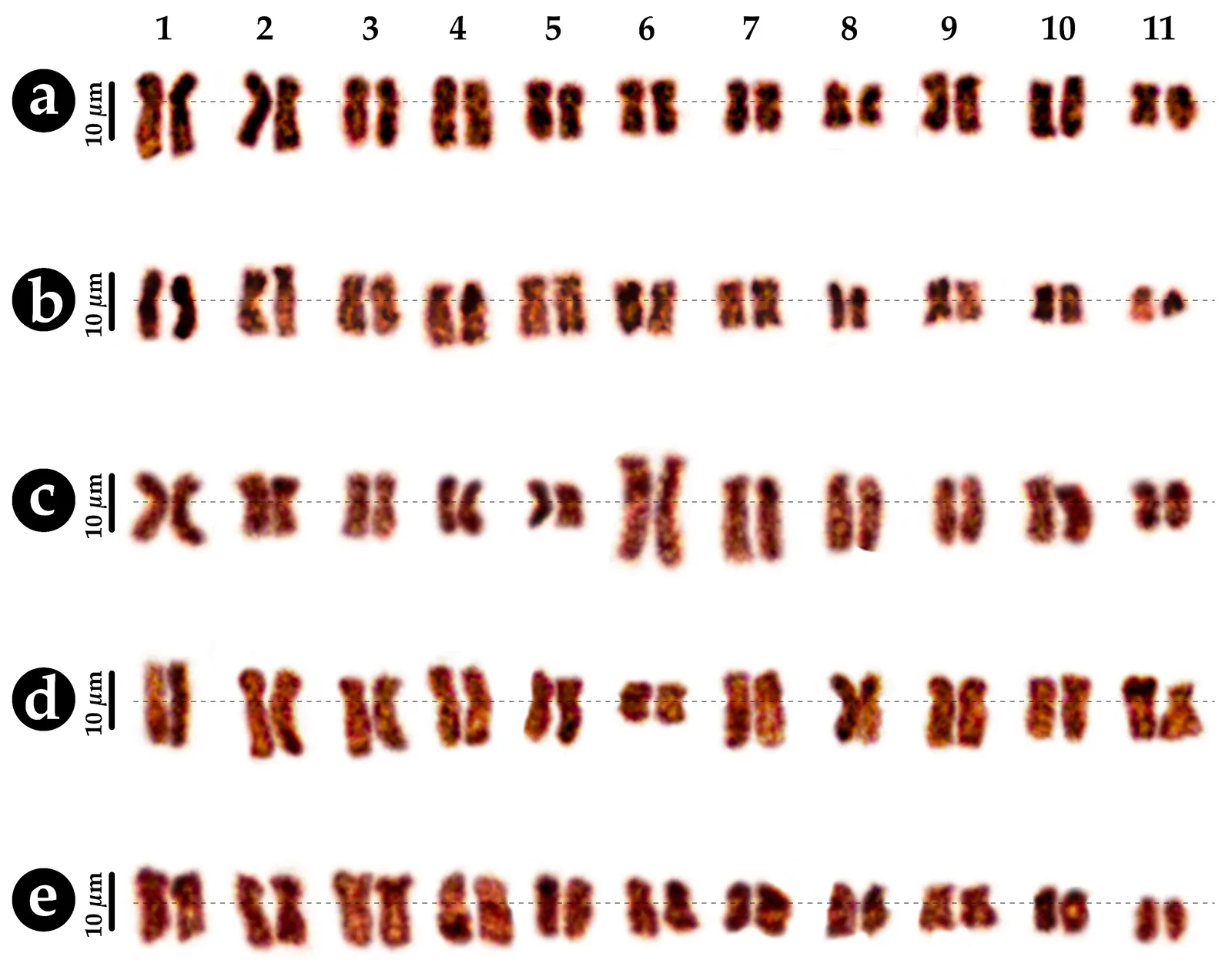
Karyograms of the five *Zingiber* species: (**a**) *Z. brachystachys*, (**b**) *Z. gramineum*, (**c**) *Z. mekongense*, (**d**) *Z. pellitum*, and (**e**) *Z. pyroglossum*. (Scale bar = 10 µm).

**Figure 4:**
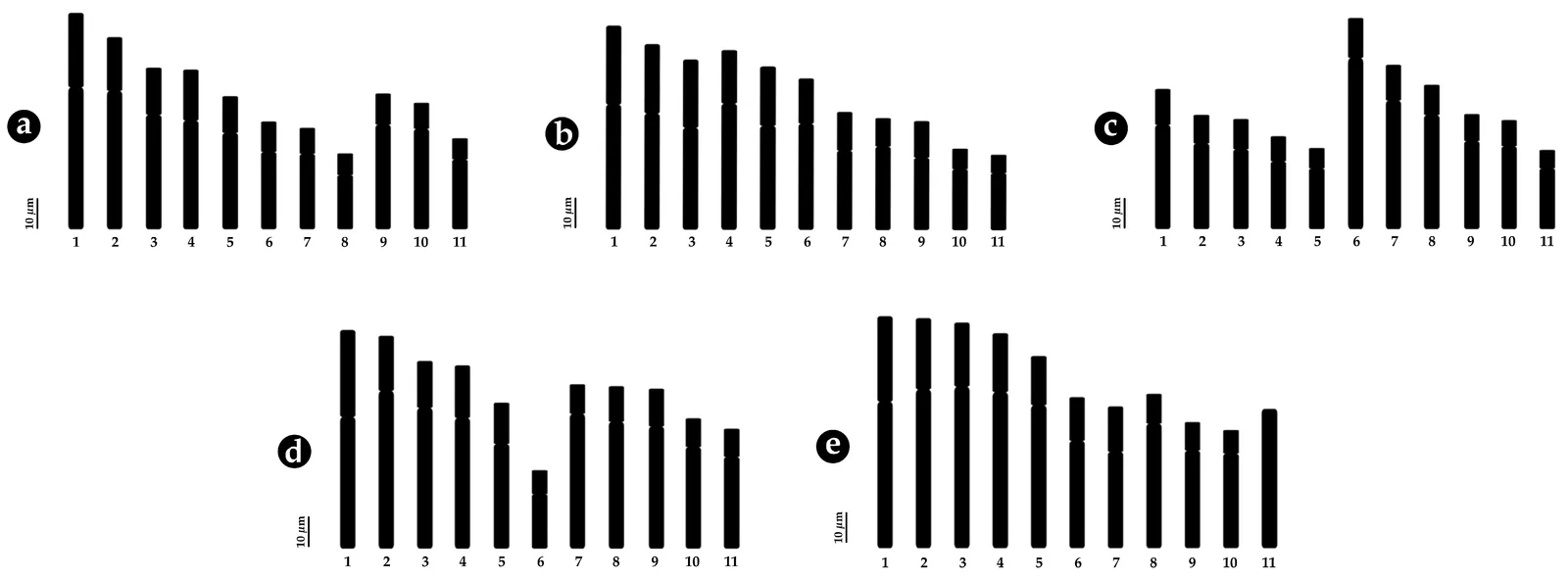
Ideograms of five *Zingiber* taxa: (**a**) *Z. brachystachys*, (**b**) *Z. gramineum*, (**c**) *Z. mekongense*, (**d**) *Z. pellitum*, and (**e**) *Z. pyroglossum*. (Scale = 10 µm).

Somatic chromosomes obtained from root-tip cells are shown in Figure 2, and the chromosome data are summarized in Table 1. Chromosome numbers were determined from 10 well-spread metaphase plates for each species. All five *Zingiber* species consistently showed a somatic chromosome number of 2*n* = 22. The chromosome numbers and detailed karyotype data reported here represent the first cytogenetic records based on material collected in Thailand for these five species. Despite having the same somatic chromosome number, the five species differed in karyotype composition. *Zingiber brachystachys* had a karyotype formula of 8sm + 3st, *Z. gramineum* 3m + 7sm + 1st, *Z. mekongense* 5sm + 6st, *Z. pellitum* 1m + 5sm + 5st, and *Z. pyroglossum* 7sm + 3st + 1t. The karyograms and corresponding ideograms are presented in Figure 3 and Figure 4, respectively.

*Zingiber brachystachys* had a somatic chromosome number of 2*n* = 22 and NF = 44 (Figure 2a and Table 1). Its chromosome complement comprised eight submetacentric and three subtelocentric chromosome pairs, corresponding to the karyotype formula 8sm + 3st (Figure 3a). Mean short-arm length (Ls) ranged from 0.30 ± 0.05 to 1.06 ± 0.06 µm, while mean long-arm length (Ll) ranged from 0.77 ± 0.05 to 2.02 ± 0.05 µm. Mean total chromosome length (LT) varied from 1.08 ± 0.09 to 3.08 ± 0.08 µm. Relative chromosome length (RL) ranged from 0.051 ± 0.004 to 0.144 ± 0.003, and the centromeric index (CI) ranged from 0.656 ± 0.014 to 0.794 ± 0.023 (Table 2). The corresponding karyogram and ideogram are shown in Figure 3a and Figure 4a, respectively.

The Thai material of *Zingiber gramineum* showed a somatic chromosome number of 2*n* = 22 and NF = 44 (Figure 2b and Table 1). Its chromosome complement comprised three metacentric, seven submetacentric, and one subtelocentric chromosome pairs, corresponding to the karyotype formula 3m + 7sm + 1st (Figure 3b). Mean short-arm length (Ls) ranged from 0.22 ± 0.01 to 0.96 ± 0.02 µm, while mean long-arm length (Ll) ranged from 0.70 ± 0.01 to 1.55 ± 0.03 µm. Mean total chromosome length (LT) varied from 0.92 ± 0.01 to 2.49 ± 0.02 µm. Relative chromosome length (RL) ranged from 0.049 ± 0.001 to 0.131 ± 0.001, and the centromeric index (CI) ranged from 0.601 ± 0.006 to 0.761 ± 0.009 (Table 3). The corresponding karyogram and ideogram are shown in Figure 3b and Figure 4b, respectively.

A chromosome complement of 2*n* = 22 with NF = 44 was observed in *Zingiber mekongense* (Figure 2c and Table 1). The complement comprised five submetacentric and six subtelocentric chromosome pairs, corresponding to the karyotype formula 5sm + 6st (Figure 3c). Mean short-arm length (Ls) ranged from 0.34 ± 0.01 to 0.74 ± 0.01 µm, while mean long-arm length (Ll) ranged from 1.11 ± 0.02 to 3.15 ± 0.02 µm. Mean total chromosome length (LT) varied from 1.46 ± 0.02 to 3.89 ± 0.02 µm. Relative chromosome length (RL) ranged from 0.058 ± 0.001 to 0.155 ± 0.001, and the centromeric index (CI) ranged from 0.724 ± 0.008 to 0.810 ± 0.002 (Table 4). The corresponding karyogram and ideogram are shown in Figure 3c and Figure 4c, respectively.

In *Zingiber pellitum*, 22 somatic chromosomes were consistently observed, corresponding to NF = 44 (Figure 2d and Table 1). The chromosome complement comprised one metacentric, five submetacentric, and five subtelocentric chromosome pairs, corresponding to the karyotype formula 1m + 5sm + 5st (Figure 3d). Mean short-arm length (Ls) ranged from 0.34 ± 0.01 to 1.25 ± 0.03 µm, while mean long-arm length (Ll) ranged from 0.77 ± 0.01 to 2.26 ± 0.02 µm. Mean total chromosome length (LT) varied from 1.11 ± 0.01 to 3.13 ± 0.04 µm. Relative chromosome length (RL) ranged from 0.044 ± 0.001 to 0.124 ± 0.001, and the centromeric index (CI) ranged from 0.601 ± 0.006 to 0.821 ± 0.004 (Table 5). The corresponding karyogram and ideogram are shown in Figure 3d and Figure 4d, respectively.

*Zingiber pyroglossum* also possessed a somatic chromosome number of 2*n* = 22 and NF = 44 (Figure 2e and Table 1). Its chromosome complement comprised seven submetacentric, three subtelocentric, and one terminal-region chromosome pair, corresponding to the karyotype formula 7sm + 3st + 1t (Figure 3e). Mean short-arm length (Ls) ranged from 0.20 ± 0.01 to 0.87 ± 0.01 µm, while mean long-arm length (Ll) ranged from 0.96 ± 0.01 to 1.65 ± 0.01 µm. Mean total chromosome length (LT) varied from 1.20 ± 0.02 to 2.37 ± 0.01 µm. Relative chromosome length (RL) ranged from 0.060 ± 0.001 to 0.119 ± 0.001, and the centromeric index (CI) ranged from 0.633 ± 0.003 to 0.877 ± 0.005 (Table 6). The corresponding karyogram and ideogram are shown in Figure 3e and Figure 4e, respectively.

### 2.2. Multivariate Analysis of Karyomorphometric Variables

**Figure 5:**
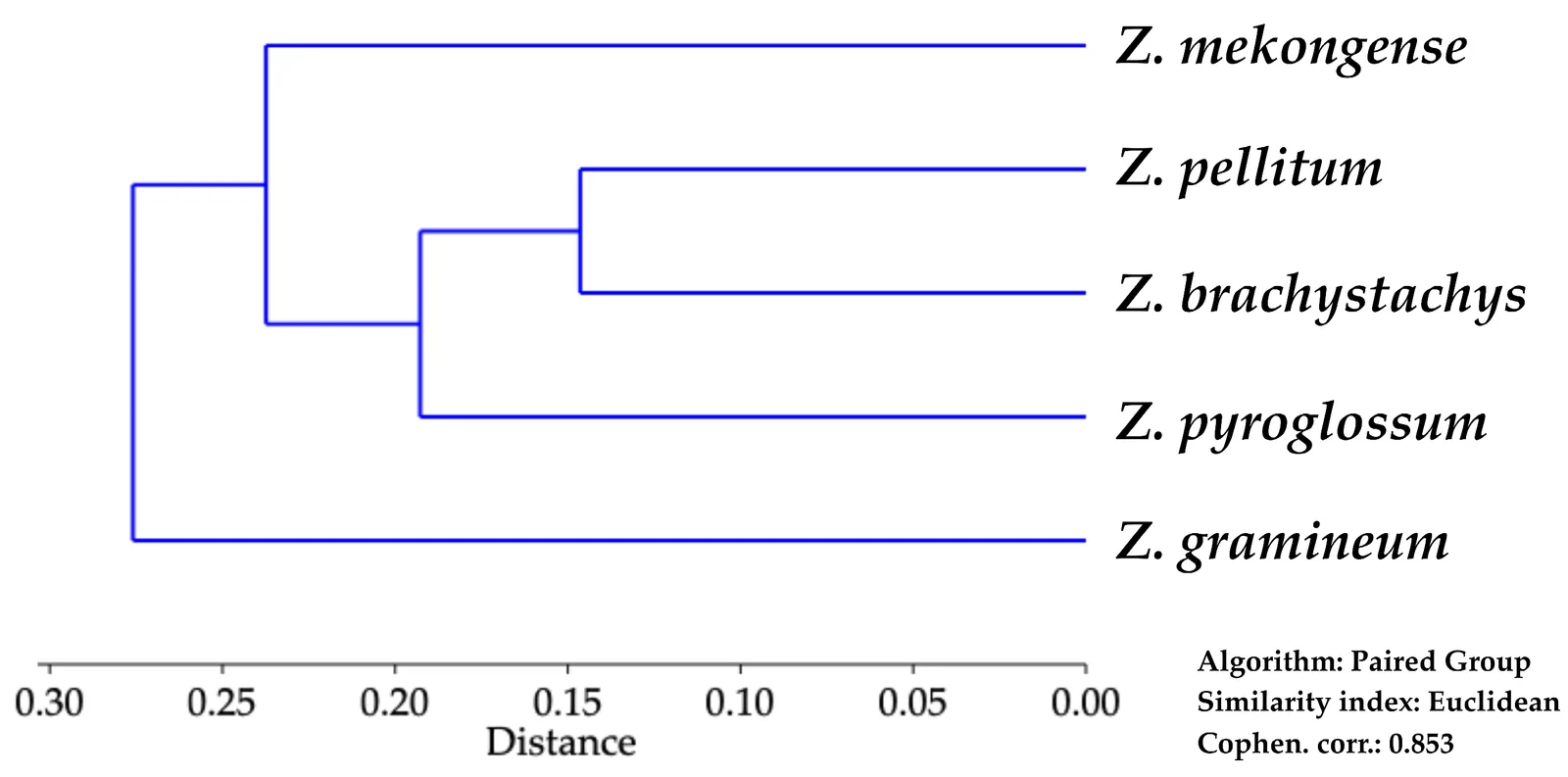
UPGMA dendrogram of five *Zingiber* species based on Euclidean distances calculated from the combined centromeric index (CI) and relative chromosome length (RL) values of 11 chromosome pairs.

Hierarchical cluster analysis based on the combined centromeric index (CI) and relative chromosome length (RL) values of the 11 chromosome pairs revealed clear karyomorphometric differentiation among the five Zingiber species (Figure 5). Zingiber pellitum and *Z. brachystachys* formed the closest cluster, joining at the lowest clustering distance. Zingiber pyroglossum subsequently joined this cluster, followed by *Z. mekongense*. In contrast, *Z. gramineum* joined the remaining taxa at the greatest distance and was therefore the most differentiated species in the combined CI–RL dataset. The cophenetic correlation coefficient of 0.853 indicated a good representation of the original distance relationships by the UPGMA dendrogram.

The clustering pattern indicates that, despite sharing the same somatic chromosome number (2*n* = 22), the five species differ in both centromere-position patterns and relative chromosome-length distributions. The close association between *Z. pellitum* and *Z. brachystachys* reflects greater similarity in their combined CI and RL profiles. *Zingiber pyroglossum* subsequently joined this cluster, followed by *Z. mekongense*, whereas *Z. gramineum* was separated from the remaining taxa at the greatest clustering distance. These results indicate that incorporating RL together with CI provides a broader representation of karyomorphometric differentiation among the five species than centromeric-index variation alone.

**Figure 6:**
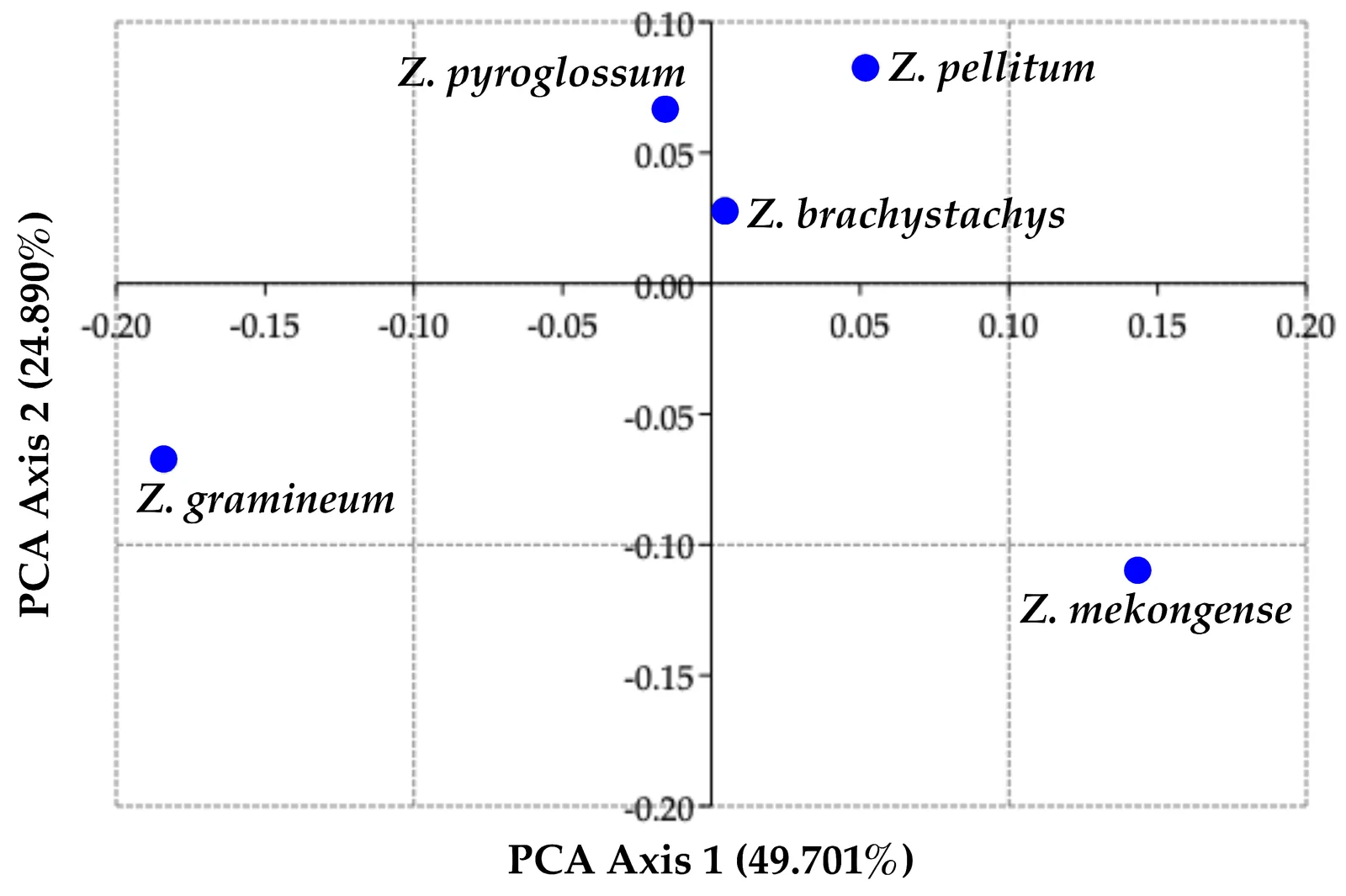
Principal component analysis (PCA) of five *Zingiber* species based on the combined centromeric index (CI) and relative chromosome length (RL) values of 11 chromosome pairs. The first two principal components explained 74.591% of the total variation (PC1 = 49.701%; PC2 = 24.890%).

Principal component analysis (PCA) based on the combined centromeric index (CI) and relative chromosome length (RL) values of the 11 chromosome pairs revealed clear differentiation among the five *Zingiber* species (Figure 6). The first principal component (PC1) accounted for 49.701% of the total variation, while the second principal component (PC2) accounted for 24.890%. Together, the first two components explained 74.591% of the total variation in the combined CI–RL dataset. *Zingiber gramineum* occupied the most negative position along PC1, whereas *Z. mekongense* occurred at the positive extreme of this axis. *Zingiber pellitum* was positioned on the positive sides of both PC1 and PC2, while *Z. pyroglossum* was characterized by a slightly negative PC1 score and a strongly positive position along PC2. Zingiber brachystachys occurred closer to the centre of the ordination. Thus, PC1 primarily distinguished *Z. gramineum* and *Z. mekongense*, whereas PC2 further separated *Z. pellitum* and *Z. pyroglossum* from taxa positioned on the negative side of this axis.

The five species occupied distinct positions in the PCA ordination. *Zingiber gramineum* was positioned at the most negative extreme of PC1 and also showed a negative PC2 score, whereas *Z. mekongense* occupied the most positive position along PC1 but was located on the negative side of PC2. *Zingiber pellitum* was positioned on the positive sides of both axes and showed the highest positive score on PC2. *Zingiber pyroglossum* was characterized by a slightly negative PC1 score and a strongly positive position along PC2, while *Z. brachystachys* occurred close to the centre of the ordination with slightly positive scores on both axes. Thus, PC1 primarily distinguished *Z. gramineum* and *Z. mekongense* at opposite extremes, whereas PC2 further differentiated *Z. pellitum* and *Z. pyroglossum* from the taxa positioned on the negative side of this axis. The PCA therefore summarized clear interspecific variation in the combined centromeric-index and relative chromosome-length profiles of the five *Zingiber* species.

### 2.3. Karyotype Asymmetry Classification

Karyotype asymmetry of the five Zingiber species was evaluated according to the classification of Stebbins [28], based on two parameters: the ratio of the longest to the shortest chromosome and the proportion of chromosome pairs with an arm ratio (AR) greater than 2:1. The longest-to-shortest chromosome ratio ranged from 1.975 in *Z. pyroglossum* to 2.852 in *Z. brachystachys*, whereas the proportion of chromosome pairs with AR > 2:1 ranged from 0.545 in *Z. gramineum* to 1.000 in *Z. mekongense* (Table 7).

Three Stebbins asymmetry categories were recognized among the five species. *Zingiber pyroglossum* was classified as 3A because 10 of its 11 chromosome pairs had AR > 2:1, while its longest-to-shortest chromosome ratio remained below 2.0. *Zingiber brachystachys*, *Z. gramineum*, and *Z. pellitum* were classified as 3B, with more than half but not all chromosome pairs showing AR > 2:1 and longest-to-shortest chromosome ratios between 2.0 and 4.0. *Zingiber mekongense* was assigned to category 4B because all 11 chromosome pairs had AR > 2:1 and its longest-to-shortest chromosome ratio also fell between 2.0 and 4.0. Among the five species examined, *Z. mekongense* therefore showed the highest Stebbins asymmetry category recorded in this study.

## 3. Discussion

The present study provides the first cytogenetic data based on material collected in Thailand for *Zingiber brachystachys*, *Z. gramineum*, *Z. mekongense*, *Z. pellitum*, and *Z. pyroglossum*. All five examined taxa showed the same somatic chromosome number, 2*n* = 22, and fundamental number, NF = 44, consistent with the chromosome number most frequently reported in Zingiber, for which *x* = 11 has generally been recognized as the basic chromosome number [14,15,16,17,18,19,20,21]. Despite this numerical uniformity, the five taxa differed in chromosome morphology and karyotype composition, with formulae of 8sm + 3st in *Z. brachystachys*, 3m + 7sm + 1st in *Z. gramineum*, 5sm + 6st in *Z. mekongense*, 1m + 5sm + 5st in *Z. pellitum*, and 7sm + 3st + 1t in *Z. pyroglossum*. A comparable pattern has been reported in other members of the genus. Omanakumari and Mathew [22] recorded 2*n* = 22 in four *Zingiber* species but found differences in centromere position and other karyotype characters, while Saensouk et al. [29] reported 2*n* = 22 and NF = 44 in four Thai *Zingiber* species with differing karyotype compositions. Together, these observations indicate that chromosome number alone provides limited discrimination among taxa with 2*n* = 22, whereas chromosome size and centromere position provide additional comparative cytogenetic characters.

Chromosome-number uniformity in the material examined here does not represent the full range of cytological variation known within *Zingiber.* For example, *Z. mioga* has been reported with different chromosome numbers from different geographical sources. Ikeda et al. [23] recorded 2*n* = 44 in Korean material and noted previous reports of 2*n* = 22 from China and 2*n* = 55 from Japan. Such records demonstrate that chromosome-number variation can occur within the genus. In the present study, however, all 10 metaphase cells examined for each taxon consistently showed 2*n* = 22. Because only one individual from one locality was investigated for each species, the present results characterize the examined voucher-linked material and should not be interpreted as representing the full range of intraspecific cytogenetic variation. Additional individuals and populations will therefore be required to evaluate the stability of these chromosome numbers and karyomorphometric patterns.

The chromosome count obtained for Thai *Z. gramineum* is particularly noteworthy because it differs from the previous report of 2*n* = 32 by Etikawati and Setyawan [30] for material originating from Bogor Botanical Garden, West Java, Indonesia. The earlier study described chromosomes generally 2 µm or less in length and predominantly metacentric, but also noted difficulties in accurately determining the morphology and dimensions of individual chromosome pairs and was unable to construct a detailed karyotype. Several non-exclusive explanations may account for the contrasting chromosome counts. The difference may represent genuine geographical variation in chromosome number, as variation among geographically separated material is known elsewhere in *Zingiber*. Alternatively, differences in the taxonomic identity of the examined material or technical difficulties associated with resolving small and overlapping chromosomes may have contributed to the discrepancy. None of these possibilities can presently be confirmed. Re-examination of Indonesian material together with additional wild populations from Thailand and other parts of the species range will be necessary to determine the basis of the contrasting chromosome counts.

The combined analysis of centromeric index (CI) and relative chromosome length (RL) provided a broader quantitative assessment of karyomorphometric differentiation among the five taxa. In the UPGMA analysis, *Z. pellitum* and *Z. brachystachys* formed the closest cluster, followed by *Z. pyroglossum* and then *Z. mekongense*, whereas *Z. gramineum* joined the remaining taxa at the greatest clustering distance. The PCA similarly distinguished the five taxa, with PC1 and PC2 accounting for 49.701% and 24.890% of the total variation, respectively, and jointly explaining 74.591%. *Zingiber gramineum* and *Z. mekongense* occupied opposite extremes of PC1, whereas *Z. pellitum* and *Z. pyroglossum* were further differentiated along PC2. The combined analysis indicates that relative chromosome-length distribution contributes information complementary to that provided by centromeric indices. Thus, CI and RL represent different components of chromosome-complement structure and, when considered together, provide a broader representation of karyomorphometric differences among the examined taxa.

Karyotype asymmetry provided an additional means of comparing chromosome complements that shared the same chromosome number. According to the Stebbins classification [28], *Z. pyroglossum* was assigned to category 3A, *Z. brachystachys*, *Z. gramineum*, and *Z. pellitum* to 3B, and *Z. mekongense* to 4B. The classification of *Z. mekongense* as 4B reflected the occurrence of AR > 2:1 in all 11 chromosome pairs, whereas *Z. pyroglossum* remained in category 3A because its longest-to-shortest chromosome ratio was below 2.0. These categories differ from those reported by Omanakumari and Mathew [22], who classified *Z. officinale* as 1A and *Z. zerumbet*, *Z. wightianum*, and *Z. macrostachyum* as 2A. The comparison demonstrates variation in chromosome-size distribution and chromosome-arm proportions among *Zingiber* species with otherwise similar chromosome numbers. However, the present data do not by themselves permit conclusions regarding the origin or evolutionary significance of these differences.

Overall, the results show that the examined *Zingiber* taxa share a common somatic chromosome number and NF but can be distinguished by chromosome morphology, relative chromosome-length distribution, centromere position, and karyotype asymmetry. The combined CI–RL analyses further demonstrate that these complementary karyomorphometric variables provide greater resolution than chromosome number or centromeric-index variation alone. Although the present sampling does not permit assessment of intraspecific variation, the voucher-linked chromosome data provide a comparative baseline for further cytogenetic investigation of *Zingiber* in Thailand.

## 4. Materials and Methods

### 4.1. Plant Materials and Sample Collection

Five species of *Zingiber* were collected from natural habitats in the Southwestern, Central, and Southeastern floristic regions of Thailand. *Zingiber brachystachys* Triboun & K.Larsen (voucher specimen: Saensouk 3900) was collected from Lop Buri Province in the Central floristic region; *Z. gramineum* Noronha ex Blume (Saensouk 3901) was collected from Kanchanaburi Province in the Southwestern floristic region; *Z. mekongense* Gagnep. (Saensouk 3902), *Z. pellitum* Gagnep. (Saensouk 3903), and *Z. pyroglossum* Triboun & K.Larsen (Saensouk 3904) were collected from Chanthaburi, Sa Kaeo, and Prachin Buri Provinces, respectively, in the Southeastern floristic region (Figure 1).

In the present study, these five taxa are referred to as rare because they were infrequently encountered during field surveys and occurred only in localized habitats within the surveyed areas. The term “rare” is used here in a local floristic sense and does not imply a threatened category under the IUCN Red List. Living plants were subsequently cultivated in a nursery at Mahasarakham University, Maha Sarakham Province, Thailand. For the cytogenetic investigation, one cultivated individual representing each species was examined. Actively growing root tips used for chromosome preparation and karyomorphometric analysis were obtained from the same individual of each species. Voucher specimens were deposited in the Vascular Plant Herbarium, Mahasarakham University (VMSU).

### 4.2. Somatic Chromosome Preparation and Karyotype Analysis

Actively growing root tips were collected from cultivated plants of the five *Zingiber* species. The root tips were pretreated with p-dichlorobenzene at 4 °C for 6 h and subsequently fixed in ethanol–acetic acid (3:1, *v*/*v*) at room temperature for 30 min. The fixed material was stored at 4 °C until chromosome preparation.

The fixed root tips were rinsed with distilled water and hydrolyzed in 1 M hydrochloric acid (HCl) at 60 °C for 5 min, followed by rinsing with distilled water. The meristematic portions of the root tips were stained with 2% aceto-orcein, squashed on microscope slides, and examined under a Zeiss Axiostar Plus light microscope (Carl Zeiss Light Microscopy, Göttingen, Germany) at 400× magnification.

For each species, 10 well-spread somatic metaphase cells obtained from root tips of the same individual were selected for chromosome counting and karyomorphometric measurements. Digital photomicrographs of the selected metaphase cells were analyzed using ImageJ software Version 1.54g (National Institutes of Health, Bethesda, MD, USA). The image scale was calibrated against the corresponding microscope scale before measurement. For each chromosome, the short-arm length (Ls) and long-arm length (Ll) were measured, and total chromosome length (LT) was calculated as Ls + Ll. Relative chromosome length (RL) and centromeric index (CI) were subsequently calculated from the chromosome measurements. Chromosomes were classified according to centromere position following Levan et al. [31], and karyotype formulae were determined for each species.

### 4.3. Karyomorphometric and Statistical Analyses

Karyomorphometric data for each species were obtained from 10 well-spread metaphase cells from a single individual. For each corresponding chromosome pair, mean values and standard deviations (SD) of Ls, Ll, LT, RL, and CI were calculated across the 10 metaphase cells.

Principal component analysis (PCA) was performed using the combined centromeric index (CI) and relative chromosome length (RL) values of the 11 chromosome pairs, resulting in 22 karyomorphometric variables for each taxon. PCA was based on the variance–covariance matrix. Hierarchical cluster analysis was performed using Euclidean distances calculated from the same combined CI–RL dataset, with clustering by the unweighted pair group method with arithmetic mean (UPGMA). The cophenetic correlation coefficient was calculated to evaluate the correspondence between the dendrogram and the original distance matrix. PCA was performed using R version 4.6.1 (R Foundation for Statistical Computing, Vienna, Austria) [32], whereas UPGMA analysis was conducted using PAST version 4.15 [33,34]. Karyotype asymmetry was additionally classified according to Stebbins [28], using the ratio of the longest to the shortest chromosome and the proportion of chromosome pairs with an arm ratio greater than 2:1.

## 5. Conclusions

This study provides the first cytogenetic data based on material collected in Thailand for *Zingiber brachystachys*, *Z. gramineum*, *Z. mekongense*, *Z. pellitum*, and *Z. pyroglossum*. All five examined taxa had a somatic chromosome number of 2*n* = 22 and NF = 44 but differed in karyotype composition, chromosome-size distribution, and centromere position. Their karyotype formulae were 8sm + 3st, 3m + 7sm + 1st, 5sm + 6st, 1m + 5sm + 5st, and 7sm + 3st + 1t, respectively. Multivariate analyses incorporating both centromeric index (CI) and relative chromosome length (RL) further differentiated their chromosome complements, with *Z. pellitum* and *Z. brachystachys* showing the closest similarity in the UPGMA analysis, whereas *Z. gramineum* was separated from the remaining taxa at the greatest clustering distance. The first two PCA components explained 74.591% of the total variation in the combined CI–RL dataset. Karyotype asymmetry was represented by Stebbins categories 3A, 3B, and 4B, with *Z. mekongense* assigned to the highest category recorded among the examined taxa. These findings demonstrate that, despite uniformity in chromosome number and NF, quantitative differences in chromosome morphology provide useful characters for distinguishing the examined chromosome complements. The present study therefore provides a baseline for broader population-level and comparative cytogenetic investigations of *Zingiber* in Thailand.

## Figures and Tables

**Table 1:** Cytological data, distribution, and voucher information for the five *Zingiber* species examined in this study.

Species	*n*	2*n*	NF	Karyotype Formula	IUCN Red List Status	Distribution Status in Thailand	Rarity Status	Floristic Region	Location (Provinces)	Voucher Specimens
*Z. brachystachys*	-	22 *	44 *	8sm + 3st *	NE	Endemic	Rare	Central	Lop Buri	Saensouk 3900 (VMSU)
*Z. gramineum*	-	22 *	44 *	3m + 7sm + 1st *	LC [25]	Native	Rare	Southwestern	Kanchanaburi	Saensouk 3901 (VMSU)
*Z. mekongense*	-	22 *	44 *	5sm + 6st *	NE	Native	Rare	Southeastern	Chanthaburi	Saensouk 3902 (VMSU)
*Z. pellitum*	-	22 *	44 *	1m + 5sm + 5st *	LC [26]	Native	Rare	Southeastern	Sa Kaeo	Saensouk 3903 (VMSU)
*Z. pyroglossum*	-	22 *	44 *	7sm + 3st + 1t *	LC [27]	Native	Rare	Southeastern	Prachin Buri	Saensouk 3904 (VMSU)

Abbreviations and notes: *n* = gametic chromosome number; 2*n* = somatic chromosome number; NF = fundamental number; m = metacentric chromosome pair; sm = submetacentric chromosome pair; st = subtelocentric chromosome pair; t = terminal-region chromosome pair; NE = Not Evaluated; LC = Least Concern; dash = not investigated. * = The cytogenetic data presented here represent the first records based on material collected in Thailand for these species.

**Table 2:** Mean short-arm length (Ls), long-arm length (Ll), total chromosome length (LT), relative length (RL) and centromeric index (CI) of *Zingiber brachystachys* 2*n* = 22.

Chro. Pair	Ls ± SD (µm)	Ll ± SD (µm)	LT ± SD (µm)	RL	CI	Chromosome Type
1	1.06 ± 0.06	2.02 ± 0.05	3.08 ± 0.08	0.144 ± 0.003	0.656 ± 0.014	Submetacentric (sm)
2	0.77 ± 0.03	1.97 ± 0.04	2.74 ± 0.05	0.128 ± 0.002	0.719 ± 0.009	Submetacentric (sm)
3	0.67 ± 0.05	1.63 ± 0.02	2.30 ± 0.05	0.108 ± 0.002	0.709 ± 0.016	Submetacentric (sm)
4	0.72 ± 0.02	1.55 ± 0.02	2.27 ± 0.03	0.106 ± 0.001	0.683 ± 0.007	Submetacentric (sm)
5	0.52 ± 0.01	1.37 ± 0.01	1.89 ± 0.01	0.088 ± 0.001	0.725 ± 0.004	Submetacentric (sm)
6	0.43 ± 0.03	1.10 ± 0.01	1.53 ± 0.03	0.072 ± 0.001	0.719 ± 0.014	Submetacentric (sm)
7	0.37 ± 0.03	1.07 ± 0.05	1.44 ± 0.06	0.067 ± 0.003	0.743 ± 0.018	Submetacentric (sm)
8	0.31 ± 0.07	0.77 ± 0.05	1.08 ± 0.09	0.051 ± 0.004	0.713 ± 0.048	Submetacentric (sm)
9	0.44 ± 0.02	1.49 ± 0.01	1.93 ± 0.02	0.090 ± 0.001	0.772 ± 0.008	Subtelocentric (st)
10	0.37 ± 0.05	1.43 ± 0.04	1.80 ± 0.06	0.084 ± 0.003	0.794 ± 0.023	Subtelocentric (st)
11	0.30 ± 0.05	1.00 ± 0.02	1.30 ± 0.05	0.061 ± 0.002	0.769 ± 0.030	Subtelocentric (st)

**Table 3:** Mean short-arm length (Ls), long-arm length (Ll), total chromosome length (LT), relative length (RL) and centromeric index (CI) of *Z. gramineum* 2*n* = 22.

Chro. Pair	Ls ± SD (µm)	Ll ± SD (µm)	LT ± SD (µm)	RL	CI	Chromosome Type
1	0.96 ± 0.02	1.53 ± 0.01	2.49 ± 0.02	0.131 ± 0.001	0.614 ± 0.005	Metacentric (m)
2	0.85 ± 0.01	1.42 ± 0.01	2.27 ± 0.01	0.120 ± 0.001	0.626 ± 0.003	Metacentric (m)
3	0.83 ± 0.02	1.25 ± 0.01	2.08 ± 0.02	0.110 ± 0.001	0.601 ± 0.006	Metacentric (m)
4	0.65 ± 0.01	1.55 ± 0.03	2.20 ± 0.03	0.116 ± 0.002	0.705 ± 0.005	Submetacentric (sm)
5	0.72 ± 0.02	1.27 ± 0.02	1.99 ± 0.03	0.105 ± 0.001	0.638 ± 0.007	Submetacentric (sm)
6	0.55 ± 0.01	1.30 ± 0.01	1.85 ± 0.01	0.098 ± 0.001	0.703 ± 0.004	Submetacentric (sm)
7	0.47 ± 0.01	0.97 ± 0.01	1.44 ± 0.01	0.076 ± 0.001	0.674 ± 0.005	Submetacentric (sm)
8	0.35 ± 0.02	1.02 ± 0.02	1.37 ± 0.03	0.072 ± 0.001	0.745 ± 0.011	Submetacentric (sm)
9	0.45 ± 0.02	0.88 ± 0.02	1.33 ± 0.03	0.070 ± 0.001	0.662 ± 0.011	Submetacentric (sm)
10	0.25 ± 0.01	0.75 ± 0.03	1.00 ± 0.03	0.053 ± 0.002	0.750 ± 0.011	Submetacentric (sm)
11	0.22 ± 0.01	0.70 ± 0.01	0.92 ± 0.01	0.049 ± 0.001	0.761 ± 0.009	Subtelocentric (st)

**Table 4:** Mean short-arm length (Ls), long-arm length (Ll), total chromosome length (LT), relative length (RL) and centromeric index (CI) of *Z. mekongense* 2*n* = 22.

Chro. Pair	Ls ± SD (µm)	Ll ± SD (µm)	LT ± SD (µm)	RL	CI	Chromosome Type
1	0.66 ± 0.01	1.92 ± 0.01	2.58 ± 0.01	0.103 ± 0.001	0.744 ± 0.003	Submetacentric (sm)
2	0.53 ± 0.02	1.57 ± 0.02	2.10 ± 0.03	0.084 ± 0.001	0.748 ± 0.008	Submetacentric (sm)
3	0.56 ± 0.02	1.47 ± 0.02	2.03 ± 0.03	0.081 ± 0.001	0.724 ± 0.008	Submetacentric (sm)
4	0.46 ± 0.03	1.24 ± 0.02	1.70 ± 0.04	0.068 ± 0.001	0.729 ± 0.013	Submetacentric (sm)
5	0.38 ± 0.01	1.11 ± 0.02	1.49 ± 0.02	0.060 ± 0.001	0.745 ± 0.006	Submetacentric (sm)
6	0.74 ± 0.01	3.15 ± 0.02	3.89 ± 0.02	0.155 ± 0.001	0.810 ± 0.002	Subtelocentric (st)
7	0.65 ± 0.01	2.37 ± 0.01	3.02 ± 0.01	0.121 ± 0.001	0.785 ± 0.003	Subtelocentric (st)
8	0.55 ± 0.02	2.10 ± 0.01	2.65 ± 0.02	0.106 ± 0.001	0.792 ± 0.006	Subtelocentric (st)
9	0.50 ± 0.03	1.62 ± 0.01	2.12 ± 0.03	0.085 ± 0.001	0.764 ± 0.011	Subtelocentric (st)
10	0.48 ± 0.03	1.52 ± 0.03	2.00 ± 0.04	0.080 ± 0.002	0.760 ± 0.012	Subtelocentric (st)
11	0.34 ± 0.01	1.12 ± 0.02	1.46 ± 0.02	0.058 ± 0.001	0.767 ± 0.006	Subtelocentric (st)

**Table 5:** Mean short-arm length (Ls), long-arm length (Ll), total chromosome length (LT), relative length (RL) and centromeric index (CI) of *Z. pellitum* 2*n* = 22.

Chro. Pair	Ls ± SD (µm)	Ll ± SD (µm)	LT ± SD (µm)	RL	CI	Chromosome Type
1	1.25 ± 0.03	1.88 ± 0.02	3.13 ± 0.04	0.124 ± 0.001	0.601 ± 0.006	Metacentric (m)
2	0.79 ± 0.04	2.26 ± 0.02	3.05 ± 0.04	0.121 ± 0.002	0.741 ± 0.010	Submetacentric (sm)
3	0.67 ± 0.02	2.01 ± 0.02	2.68 ± 0.03	0.106 ± 0.001	0.750 ± 0.006	Submetacentric (sm)
4	0.75 ± 0.03	1.87 ± 0.02	2.62 ± 0.04	0.104 ± 0.001	0.714 ± 0.008	Submetacentric (sm)
5	0.59 ± 0.02	1.50 ± 0.02	2.09 ± 0.03	0.083 ± 0.001	0.718 ± 0.007	Submetacentric (sm)
6	0.34 ± 0.01	0.77 ± 0.01	1.11 ± 0.01	0.044 ± 0.001	0.694 ± 0.007	Submetacentric (sm)
7	0.42 ± 0.01	1.93 ± 0.01	2.35 ± 0.01	0.093 ± 0.001	0.821 ± 0.004	Subtelocentric (st)
8	0.51 ± 0.03	1.81 ± 0.01	2.32 ± 0.03	0.092 ± 0.001	0.780 ± 0.010	Subtelocentric (st)
9	0.54 ± 0.04	1.75 ± 0.04	2.29 ± 0.06	0.091 ± 0.002	0.764 ± 0.014	Subtelocentric (st)
10	0.41 ± 0.01	1.45 ± 0.01	1.86 ± 0.01	0.074 ± 0.001	0.780 ± 0.004	Subtelocentric (st)
11	0.40 ± 0.01	1.31 ± 0.01	1.71 ± 0.01	0.068 ± 0.001	0.766 ± 0.005	Subtelocentric (st)

**Table 6:** Mean short-arm length (Ls), long-arm length (Ll), total chromosome length (LT), relative length (RL) and centromeric index (CI) of *Z. pyroglossum* 2*n* = 22.

Chro. Pair	Ls ± SD (µm)	Ll ± SD (µm)	LT ± SD (µm)	RL	CI	Chromosome Type
1	0.87 ± 0.01	1.50 ± 0.01	2.37 ± 0.01	0.119 ± 0.001	0.633 ± 0.003	Submetacentric (sm)
2	0.73 ± 0.01	1.62 ± 0.01	2.35 ± 0.01	0.118 ± 0.001	0.689 ± 0.003	Submetacentric (sm)
3	0.65 ± 0.01	1.65 ± 0.01	2.30 ± 0.01	0.116 ± 0.001	0.717 ± 0.003	Submetacentric (sm)
4	0.60 ± 0.01	1.60 ± 0.01	2.20 ± 0.01	0.111 ± 0.001	0.727 ± 0.004	Submetacentric (sm)
5	0.50 ± 0.01	1.46 ± 0.01	1.96 ± 0.01	0.099 ± 0.001	0.745 ± 0.004	Submetacentric (sm)
6	0.44 ± 0.01	1.10 ± 0.01	1.54 ± 0.01	0.078 ± 0.001	0.714 ± 0.005	Submetacentric (sm)
7	0.46 ± 0.01	0.98 ± 0.01	1.44 ± 0.01	0.073 ± 0.001	0.681 ± 0.005	Submetacentric (sm)
8	0.30 ± 0.01	1.27 ± 0.01	1.57 ± 0.01	0.079 ± 0.001	0.809 ± 0.005	Subtelocentric (st)
9	0.29 ± 0.02	1.00 ± 0.01	1.29 ± 0.02	0.065 ± 0.001	0.775 ± 0.012	Subtelocentric (st)
10	0.24 ± 0.02	0.96 ± 0.01	1.20 ± 0.02	0.060 ± 0.001	0.800 ± 0.013	Subtelocentric (st)
11	0.20 ± 0.01	1.42 ± 0.01	1.62 ± 0.01	0.082 ± 0.001	0.877 ± 0.005	Terminal-region (t)

**Table 7:** Karyotype asymmetry classification of the five *Zingiber* species according to Stebbins [28].

Species	Longest Chromosome (µm)	Shortest Chromosome (µm)	Longest/ Shortest Ratio	Chromosome Pairs with AR > 2:1	Proportion with AR > 2:1	Stebbins Category
*Z. brachystachys*	3.08	1.08	2.852	10/11	0.909	3B
*Z. gramineum*	2.49	0.92	2.707	6/11	0.545	3B
*Z. mekongense*	3.89	1.46	2.664	11/11	1.000	4B
*Z. pellitum*	3.13	1.11	2.820	10/11	0.909	3B
*Z. pyroglossum*	2.37	1.20	1.975	10/11	0.909	3A

**Notes:** AR = arm ratio, calculated as long-arm length/short-arm length (Ll/Ls). Stebbins categories were determined from the ratio of the longest to the shortest chromosome and the proportion of chromosome pairs with an arm ratio > 2:1. Classification follows Stebbins [28].

## Data Availability

The original contributions presented in this study are included in the article. Further inquiries can be directed to the corresponding author.
